# Pharmacological Assessment of the Medicinal Potential of *Acacia mearnsii* De Wild.: Antimicrobial and Toxicity Activities

**DOI:** 10.3390/ijms13044255

**Published:** 2012-03-30

**Authors:** Olufunmiso O. Olajuyigbe, Anthony J. Afolayan

**Affiliations:** Phytomedicine Research Centre, Department of Botany, University of Fort Hare, Alice 5700, South Africa; E-Mail: funmijuyigbe12@yahoo.com

**Keywords:** *Acacia mearnsii*, antimicrobial activity, bactericidal, cytotoxic effects, extract

## Abstract

*Acacia mearnsii* De Wild. (Fabaceae) is a medicinal plant used in the treatment of microbial infections in South Africa without scientific validation of its bioactivity and toxicity. The antimicrobial activity of the crude acetone extract was evaluated by both agar diffusion and macrobroth dilution methods while its cytotoxicity effect was assessed with brine shrimp lethality assay. The study showed that both bacterial and fungal isolates were highly inhibited by the crude extract. The MIC values for the gram-positive bacteria (78.1–312.5) μg/mL, gram-negative bacteria (39.1–625) μg/mL and fungal isolates (625–5000) μg/mL differ significantly. The bacteria were more susceptible than the fungal strains tested. The antibiosis determination showed that the extract was more (75%) bactericidal than bacteriostatic (25%) and more fungicidal (66.67%) than fungistatic (33.33%). The cytotoxic activity of the extract was observed between 31.25 μg/mL and 500 μg/mL and the LC_50_ value (112.36 μg/mL) indicates that the extract was nontoxic in the brine shrimp lethality assay (LC_50_ > 100 μg/mL). These results support the use of *A. mearnsii* in traditional medicine for treatment of microbial infections. The extract exhibiting significant broad spectrum antimicrobial activity and nontoxic effects has potential to yield active antimicrobial compounds.

## 1. Introduction

In Africa, the use of remedies derived from plants in traditional health practices is common and widespread [1] even before the introduction of antibiotics and other modern drugs [2]. While more than 80% of the world’s population still depends upon the traditional medicines for various diseases [3,4], Patel and Coogan [5] indicated that natural products have been used worldwide for medicinal purposes for thousands of years. Being nontoxic and easily affordable, today, there has been resurgence in the consumption and demand for medicinal plants [6] resulting from bacterial resistance to currently used antibiotics becoming a public health concern [7], rising costs of prescription drugs and the bioprospecting of new plant-derived drugs [8]. Though only 5–15% of the estimated 250,000 higher plants in the world [9] have been studied for a potential therapeutic value [10] and represent a potential source of new anti-infective agents [11,12], a large number remains to be investigated since the search for antimicrobial agents is largely concentrated on lower plants, fungi and bacteria.

Since the development of new compounds and antimicrobial agents for the treatment of microbial infections has recently become of increasing interest [13], the trend of using natural products has increased and the active plant extracts are frequently screened for new drug discoveries [14]. The effects of plant extracts on microorganisms have been studied by a very large number of researchers in different parts of the world [15–17]. The presence of antibacterial, antifungal and other biological activities have also been demonstrated in extracts of different plant species used in traditional medicine practices [18–20]. On this basis, several reports have indicated that antimicrobial activity of crude plant extracts and the bioassay-guided fractionation of those extracts yielded active principles [21,22].

Although there is a growing interest in correlating the phytochemical constituents of a medicinal plant with its pharmacological activity [23,24], the toxicological effects of most of these crude extracts are often overlooked based on the facts that plant medicines have better compatibility with the human body and produce fewer side effects [25]. However, to forestall adverse effects, sometimes life-threatening, allegedly arising consequential to taking herbal products or traditional medicines [26,27], cytotoxicity testing, an integral component of the biological evaluation of pharmacologically important materials and an essential part of standard screening procedures, becomes essential. Since toxicological evaluation of a plant extract seeks to determine its possible collateral effects to ensure the safety of its use, brine shrimp larvae, being sensitive to toxic substances, are commonly used for toxicity assays in pharmacology. The ratio between dead larvae (no motility) and living larvae (high motility) in comparison to a control without any toxic substance is used to estimate the toxicity of the test substances [28].

*Acacia* is a pantropical and subtropical genus with species abundant throughout Australia, Asia, Africa and America. The plant *Acacia mearnsii* De Wild. (Fabaceae), previously known as *Racosperma mearnsii* [29] and commonly known as Black Wattle tree, is a short medium lived woody perennial and spreading tree, about 15 m high with smooth and greenish-brown bark on young branches which are blackish and rough on trunk. The young branchlets are downy. While it is widespread and common in lowlands, open forest, heathy woodland and on cleared land, particularly on dry, shallow soils [30], it grows in open forest, woodland or tussock grassland, in gullies or on hillsides, in sandy or gravelly clay soils [31]. It has inflorescence globular with 20–30 tiny pale yellow flowers. Pods, dark brown to black in color, are more or less straight, 5–10 cm long, 5–8 mm wide and strongly constricted between seeds [30]. Though little is known of the pharmacological importance of this plant, its phytochemical screening showed that the total phenolic content correlated well with the antioxidant activity of the extracts [32]. Since this plant is ethobotanically relevant in the treatment of microbial infections locally and scientific report on its pharmacological importance is limited, this study was aimed at assessing the antimicrobial and toxicity activities of the crude acetone stem bark extract of *A. mearnsii in vitro* to justify its ethnotherapeutic usage.

## 2. Results and Discussion

In this study, the antibacterial, antifungal and cytotoxicity activity of the crude acetone extract of the *A. mearnsii* was determined against twelve bacterial strains, twelve fungal isolates and brine shrimps respectively. The crude extract showed varied degrees of antibacterial activity against all bacteria tested (Table 1).

Though both gram-positive and gram-negative were highly inhibited, the crude extract was most active against *Proteus vulgaris* CSIR 0030 inhibited at a minimum inhibitory concentration (MIC) of 39.1 μg/mL in comparison to other tested isolates. The MIC values for the gram-positive bacteria ranged 78.1–312.5 μg/mL while those of gram-negative bacteria ranged from 39.1–625 μg/mL (Table 2). The result of the agar diffusion and macrobroth dilution assay are complementary. Results showed susceptibility patterns of the bacteria dependent on extract concentration. At the highest concentration (2000 μg/mL) of extract, the inhibition zones for all the bacteria ranged between 19 and 35 ± 1.0 mm. While *Proteus vulgaris* CSIR 0030 with the least MIC value (39.1 μg/mL) had highest inhibition zones (35 ± 1.0 mm) in comparison to those of other isolates, *Staphylococcus aureus* OK_1_ with the following lowest MIC value (78.1 μg/mL) had the least zone (19 ± 1.0 mm), and *Escherichia coli* (ATCC 25922) and *Serratia marcescens* (ATCC 9986) with the highest MIC value (625 μg/mL) had intermediate zones of inhibition (22 and 27 ± 1.0 mm, respectively). With the exception of *Proteus vulgaris* KZN, *Staphylococcus aureus* OK_1_, *Enterococcus faecalis* KZN, *Salmonella typhi* (ATCC 13311) and *Pseudomonas aeruginosa* (ATCC 19582), other bacteria had inhibition zones at the least concentration (625 μg/mL) tested.

The antifungal activity of the extract showed that the fungal isolates were susceptible at varied concentrations. The MIC values ranged 625–5000 μg/mL. The asexually reproducing fungi exhibited higher MIC values than the non-spore forming fungi (Table 3). While the MBC values for the bacterial isolates ranged between 78.1 μg/mL and 625 μg/mL, the fungal MFC values ranged between 625 μg/mL and 20,000 μg/mL. Mechanism of antibiosis determination showed that the extract was more (75%) bactericidal than being bacteriostatic (25%) and more fungicidal (66.67%) than being fungistatic (33.33%). While the bacteria were more susceptible to the erythromycin, used as control, with zones of inhibition, except for *Enterococcus faecalis* KZN, ranging from 13 ± 1.0 mm to 38 ± 1.0 mm and MIC values ranging 0.048–12.5 μg/mL, the crude extract was not found to be ineffective against the microbial isolates test. Comparatively, the bacterial strains were more susceptible than the fungal strain.

Interacting the extract and brine shrimps at the concentrations used for the antimicrobial assay for the plant resulted in the death of the shrimps within 15 min. The assay was repeated at low concentrations ranging between 0.9765 μg/mL and 500 μg/mL. Mortality of the brine shrimps was noticed in the experimental group but the control group remained unchanged at the same time. The number of surviving brine shrimps in each vial was counted and the results were noted. The percent of mortality of the shrimp was calculated for every concentration of the test sample. The cytotoxic activity of the extract was observed between 31.25 μg/mL and 500 μg/mL. At 500 μg/mL, all the brine shrimps were killed and none was killed at 31.25 μg/mL after 24 h incubation period (Table 4). The mortality rate of the brine shrimps was found to increase with the increase in concentration of the sample. It is evident from the results of the brine shrimp lethality assay that the crude extract with the LC_50_ being 112.36 μg/mL having the highest levels of toxicity (100%) death at 500 μg/mL was non toxic (LC_50_ > 100 μg/mL).

Resulting from the global increase in microbial resistance is a need to assess the antimicrobial activity of several medicinal plants with increased spectrum, potency and novel mechanisms of action which may be systemically active and offer new hope for improved therapeutic outcomes. Consequently, in addition to the significant antimicrobial activity exhibited by *A. mearnsii*, the varied degree of antimicrobial activity of the extract could be due to the nature and level of antimicrobial agents present in the plant, their mode of action and the typical differences in the microbial cell walls between the strains [33] as well as the synergistic effects of different phytochemicals present in the plant. The antimicrobial activity at relatively minimal concentrations of the extract could be attributed to the active phytochemical compounds present in the extract at different concentrations and potent enough to inhibit or kill microbial agents. Though there is a dearth of scientific reports on the pharmacological importance of this plant, Olajuyigbe and Afolayan [34] earlier indicated that its aqueous and ethanolic extracts exhibited significant antibacterial activities. The pronounced antibacterial effect of the extract in both gram-negative and gram-positive bacteria may be attributed to its ability to damage the different cell walls to allow the active compounds to adsorb, diffuse, penetrate and interact with the affected target sites as earlier indicated by Olajuyigbe and Afolayan [17]. Furthermore, the significant multifarious effects of the extract on the fungal isolates could be due to the differences in their morphology, disruption of membrane [35] or cell wall integrity [36], inhibition of mycelia growth [37], high potential to block morphogenetic transformation [38], indirect inhibition of cell wall synthesis [39] and spore germination [40]. Hence, the degree of the fungicidal effects of the extract depends on its ability to significantly cause either, or all, of these processes.

The *in vivo* brine shrimp lethality test is a simple way of screening and fractionating physiologically active plant extract. It is based on whether the brine shrimp are dead or alive at the end of the test or on the ability to kill laboratory-cultured *Artemia nauplii* [41]. The LC_50_ (112.36 μg/mL) of this extract is in agreement with the earlier report of [42] which indicated that several extracts containing physiological active principles derived from natural products has LC_50_ ≤ 1000 μg/mL using brine shrimp bioassay. However, in agreement with Moshi *et al.* [43], the crude acetone extract was nontoxic.

Despite a great lack of investigations linking phytochemical constituents, pharmacology and toxicological activity of many medicinal plants used in ethnomedicine, the pharmaceutical industry is moving away from drug discovery or screening towards medicinal plant materials which have become the subject of public attention. In view of the fact that there is a relationship between the pharmacological activity and toxicity of natural products from plants, the degree of antimicrobial activity and toxicity exhibited by *A. mearnsii* show a good relationship. While Schmitz *et al.* [44] indicated that the compounds that showed activity in the brine shrimp could be associated to some extent to the potency of the pharmacologically active principles in natural products, the varied antimicrobial activity exhibited by this extract may be attributed to its toxicity level resulting from the synergistic activity of the various phytochemicals present in the plant. An increase in the toxicity of the plant could possibly result in higher antimicrobial effects.

In addition, the significance of the inhibitory, bactericidal and fungicidal activities of this extract may not be underestimated. Since *in vitro* antimicrobial susceptibility testing assesses the relative susceptibility of microbial pathogens to selected therapeutic agents to optimize treatment of infections in clinical settings, determination of the microbicidal activity of these antimicrobial agents against an infecting organism may be useful in guiding therapy for serious infections especially when the host immune defense is compromised. The high percentages of the bactericidal and fungicidal activity activities of this extract at high concentrations showed the potentials of its active compounds to minimize the spread of an infecting organism from the site of infection and kill invading pathogens. In view of the fact that systemically active antimicrobial agents with increased spectrum and potency may offer new hope for improved therapeutic outcomes in both competent and immunocompromised individuals, a “cidal” regimen [45–48], for which *Acacia mearnsii* is an indicator, would be preferable. This, however, may justify the ethnotherapeutic relevance of this plant in the treatment of microbial infections in South Africa.

## 3. Experimental Section

### 3.1. Collection of Plant Material

The bark materials of *A. mearnsii* De Wild were collected in August 2010, from the plant growing within the University of Fort Hare campus in Alice, South Africa. The plant was authenticated in the Department of Botany and a voucher specimen was prepared and deposited in the Griffen Herbarium of the University.

### 3.2. Extract Preparation

The bark sample was air-dried at room temperature, pulverized in a mill (Christy Lab Mill, Christy and Norris Ltd.; Process Engineers, Chelmsford, UK) and stored in a sterile air-tight container for further use. The extract of the bark was prepared in accordance to the description of Basri and Fan [49]. About 100 g of the pulverized sample was steeped in 500 mL of acetone for 72 h with shaking (Stuart Scientific Orbital Shaker, UK). The plant material was extracted for two other consecutive times. The extracts were combined, filtered through Whatman No. 1 filter paper and concentrated under reduced pressure at 40 °C using a rotary evaporator (Laborarota 4000 efficient, Heldolph, Germany). The crude extract collected was allowed to dry at room temperature to a constant weight of 21.4 g. The extract was redissolved in acetone to the required concentrations for the bioassay analysis.

The reconstituted crude acetone extract solution was sterilized by filtering through 0.45 μm membrane filter. The extract was tested for sterility after membrane filtration by introducing 2 mL of the extract into 10 mL of sterile nutrient broth and incubated at 37 °C for 24 h. A sterile extract was indicated by the absence of turbidity in the broth after the incubation period.

### 3.3. Test Organisms

The bacterial isolates used in this study included *Escherichia coli* (ATCC 25922), *Bacillus cereus* (ATCC 10702), *Proteus vulgaris* KZN, *Serratia marcescens* (ATCC 9986), *Pseudomonas aeruginosa* (ATCC 19582), *Enterococcus faecalis* KZN, *Klebsiella pneumoniae* (ATCC 10031), *Proteus vulgaris* CSIR 0030, *Bacillus pumilus* (ATCC 14884), *Klebsiella* pneumoniae KZN, *Staphylococcus aureus* OK_1_ and *Salmonella typhi* (ATCC 13311). The fungal isolates included *Candida krusei*, *Candida albicans*, *Candida rugosa*, *Candida glabrata* (ATCC 2001), *Absidia corymbifera*, *Fusarium sporotrichioides*, *Trichophyton tonsurans*, *Trichophyton mucoides* (ATCC 201382), *Penicillium notatum*, *Aspergillus niger*, *Aspergillus terreus* and *Aspergillus flavus*. These organisms were obtained from the Department of Biochemistry and Microbiology, University of Fort Hare, Alice, South Africa. The organisms were maintained on nutrient broth, nutrient agar (Biolab), potato dextrose agar and sabouraud dextrose broth. The antibacterial assays were carried out using Mueller Hinton II agar and broth (Biolab). The antifungal assays were carried out using sabouraud dextrose agar and broth.

### 3.4. Preparation of Inocula

For the bacterial inoculums preparation, the inoculum of each test bacterial strains was prepared using the colony suspension method [50]. Colonies picked from 24 h old cultures grown on nutrient agar were used to make suspensions of the test organisms in saline solution to give an optical density of approximately 0.1 at 600 nm. The suspension was then diluted 1:100 by transferring 0.1 mL of the bacterial suspension to 9.9 mL of sterile nutrient broth before use. The density of bacterial suspension for susceptibility test was finally determined by comparison with 0.5 McFarland standard of Barium sulphate solution [51].

For the fungal inoculums preparation, spore suspension for fungal bioassay was prepared according to the procedure of Murugan *et al.* [52] as modified. Briefly, 1 cm^2^ of seven day old spore producing cultures was dropped in sterile distilled water and vortexed for 30 s to release the fungal spores. The spore density of each fungus was adjusted with spectrophotometer (A_595nm_) to obtain a final concentration of approximately 10^5^ spores/mL. For the *Candida* spp., the inocula were prepared by adding 1 mL of overnight Candida cultures to 9 mL of sabouraud dextrose broth to yield 10^4^ colony forming units (CFU) per microliter of the inoculum.

### 3.5. Antibiotic Susceptibility Testing—Agar Diffusion Method

The antibiotic susceptibility testing was determined using the modified Kirby-Bauer diffusion technique [51] by swabbing the Mueller-Hinton agar (MHA) (Oxoids, UK) plates with the resultant saline suspension of each strain. Wells were then bored into the agar medium with heat sterilized 6 mm cork borer. The wells were filled with 100 μL of different concentrations (625 μg/mL, 12,500 μg/mL, 2500 μg/mL, 5000 μg/mL, 10,000 μg/mL and 20,000 μg/mL) of the crude extract and 50 μg/mL of erythromycin antibiotic taking care not to allow spillage of the solutions onto the agar surface. The determinations were done in duplicates. After 24 h of incubation, the diameter of the inhibition zones of the extract and the antibiotic was measured and interpreted using the CLSI zone diameter interpretative standards [53].

### 3.6. Macrobroth Dilution for Minimum Inhibitory Concentration (MIC)

Minimum inhibitory concentration (MIC) of the extract defined as the lowest concentration which resulted in maintenance or reduction of inoculums viability [54] was determined by serial tube dilution technique [55] against the bacterial and fungal isolates. For antibacterial assay, different concentrations of the extract ranging from 20 μg/mL to 10,000 μg/mL were prepared by serial dilutions in Mueller Hinton broth medium. Different concentrations of erythromycin (0.0122–50 μg/mL), used as positive control, were also prepared by serial dilution in Mueller Hinton broth. The tubes were inoculated with 100 μL of each of the bacterial strain. Blank Mueller Hinton broth was used as negative control.

For the antifungal assay, different concentrations of the extract ranging between 19.53 μg/mL and 40,000 μg/mL were prepared in sabouraud dextrose broth by serial dilutions. Each broth concentration was inoculated with 100 μL of the prepared fungal spores’ solution. Two control tubes were included: one with spores and broth but no plant extract and one with broth and plant extract but no spores. The bacterial containing tubes were incubated aerobically at 37 °C for 24 h. The fungal containing tubes were incubated at 27 °C for 3–5 days. The first tube in the series with no visible growth after incubation period was taken as the MIC.

### 3.7. Determination of Minimum Bactericidal and Fungicidal Concentrations (MBC/MFC)

The MBC and MFC assays were carried out as described by Cheesbrough [56]. Here, fresh nutrient agar and sabouraud dextrose agar plates were inoculated with one loopful of culture taken from each of the first five broth cultures that showed no growth in the MIC tubes. While MBC assay plates were incubated for at 37 °C for 24 h, MFC assay plates were incubated at 25 °C for 3–5 days. After the incubation periods, the lowest concentration of the extract that did not produce any bacterial or fungal growth on the solid medium was regarded as MBC and MFC values for this extract [57]. This observation was matched with the MIC test tube that did not show evidence of growth after 48 h of incubating the bacteria or spore germination for the fungi after five days of incubation.

### 3.8. Determination of Mechanisms of Antibiosis (Bactericidal or Bacteriostatic)

The mechanism of antibiosis of the extracts was calculated using the ratio of MBC/MIC (MFC/MIC) or MIC_index_ as described by Shanmughapriya *et al.* [58] to elucidate whether the observed antibacterial effects were bactericidal, fungicidal, bacteriostatic or fungistatic. When the ratio of MBC/MIC or MFC/MIC was ≤2.0, the extract was considered bactericidal or otherwise bacteriostatic. If the ratio is ≥16.0, the extract was considered ineffective.

### 3.9. Brine Shrimp Lethality Test

The brine shrimp lethality test using the larvae of brine shrimp nauplii, *Artemia salina* L. was carried out using the standard procedure [42,59]. For the extract sample, 4000 μg of the crude acetone extract was initially dissolved in 1 mL of pure dimethyl sulfoxide (DMSO) to make the extract hydrophilic after which 3 mL of sterile distilled water was added to get a concentration of 1000 μg/mL of the extract used as a stock solution. Different concentrations (0.9765–500 μg/mL) of the extract were prepared from the stock solution by serial tube dilution technique in different vials. Ten nauplii were transferred into each vial using Pasteur pipettes and were not given food because hatched brine shrimp can survive for up to 48 h without food [60] as they still feed on their yolk-sac [28]. The control vials were prepared using DMSO only and the experiment was replicated three times. After 24 h of incubation, the vials were examined, the numbers of survivors in each vial were counted and percentages of deaths were calculated. Larvae were considered dead if they did not exhibit any observable movement during several seconds of observation. The extract is regarded as non-toxic if its LC_50_ is greater than 100 μg/mL in the brine shrimp lethality assay [43]. The mean mortality percentage and LC_50_ (lethal concentration for 50% of the population) were determined using statistical analysis and the graph of Logarithm of concentration against percent lethality [61].

## 4. Conclusions

The results show that the acetone extract of *A. mearnsii* had a significant antimicrobial activity and no toxic effects on the brine shrimps. This activity may indicate the medicinal potential, suggests the broad spectrum antimicrobial potential and validates the popular use of this plant in traditional medicine for the treatment of microbial infections by rural communities in South Africa since the extract was regarded as being nontoxic on brine shrimps.

## Figures and Tables

**Table 1:** Bacterial susceptibility to different concentrations of acetone stem bark extract of *A. mearnsii*.

Tested bacterial isolates	Average inhibition zones produced by 100 μL of each concentration of antibacterial agents (±1.0 mm)						

	Erythromycin	Crude acetone extract of *A. mearnsii*					
	50	20,000	10,000	5000	2500	1250	625
	μg/mL						

*Proteus vulgaris* KZN	32	20	17	15	15	13	0
*Staphylococcus aureus* OK_1_	31	19	14	0	0	0	0
*Enterococcus faecalis* KZN	0	20	16	14	0	0	0
*Klebsiella pneumoniae* KZN	14	22	20	18	16	15	14
*Proteus vulgaris* CSIR 0030	38	35	33	27	23	20	19
*Bacillus cereus* (ATCC 10702)	30	24	22	20	17	16	14
*Escherichia coli* (ATCC 25922)	13	22	20	18	16	15	15
*Bacillus pumilus* (ATCC 14884)	16	22	19	18	18	18	16
*Salmonella typhi* (ATCC 13311)	13	27	24	20	16	14	0
*Serratia marcescens*(ATCC 9986)	18	27	24	22	21	20	18
*Klebsiella pneumoniae* (ATCC 10031)	30	22	20	18	17	16	15
*Pseudomonas aeruginosa* (ATCC 19582)	37	20	18	16	14	13	0

**Table 2:** Degree of antibacterial activities of acetone stem bark extract of *A. mearnsii*.

Tested bacterial isolates	Erythromycin	*A. mearnsii*		

	MIC (μg/mL)	MIC (μg/mL)	MBC (μg/mL)	MIC_index_
*Proteus vulgaris* KZN	12.5	312.5	625	2
*Staphylococcus aureus* OK_1_	0.1953	78.1	78.1	1
*Enterococcus faecalis* KZN	12.5	156.3	625	4
*Klebsiella pneumoniae* KZN	12.5	312.5	625	2
*Proteus vulgaris* CSIR 0030	0.048	39.1	156.3	4
*Bacillus cereus* (ATCC 10702)	0.0977	156.3	312.5	2
*Escherichia coli* (ATCC 25922)	0.3906	625	625	1
*Bacillus pumilus* (ATCC 14884)	12.5	312.5	312.5	1
*Salmonella typhi* (ATCC 13311)	6.25	156.3	156.3	1
*Serratia marcescens* (ATCC 9986)	3.125	625	625	1
*Klebsiella pneumoniae* (ATCC 10031)	0.09765	156.3	156.3	1
*Pseudomonas aeruginosa* (ATCC 19582)	0.1953	156.3	625	4

**Table 3:** Antifungal activities of acetone stem bark extract of *A. mearnsii*.

Tested fungal isolates	Antifungal activities of *A. mearnsii*		

	MIC (μg/mL)	MFC (μg/mL)	MIC_index_
*Candida krusei*	1250	2500	2
*Candida albicans*	625	1250	2
Candida rugosa	625	625	1
*Aspergillus niger*	5000	20,000	4
*Aspergillus terreus*	5000	20,000	4
*Aspergillus flavus*	5000	20,000	4
*Penicillium notatum*	5000	20,000	4
*Absidia corymbifera*	625	625	1
*Fusarium sporotrichioides*	625	625	1
*Trichophyton tonsurans*	2500	5000	2
*Candida glabrata* (ATCC 2001)	625	625	1
*Trichophyton mucoides* (ATCC 201382)	2500	5000	2

**Table 4:** Cytotoxicity effects of acetone stem bark extract of *A. mearnsii* on brine shrimps.

Conc. of extract (μg/mL)	Test 1	Test 2	Test 3	Av. No. of shrimp alive (Sample)	Av. No. of shrimp alive (Control)	% Mortality	Log. of conc.	LC_50_ μg/mL
31.25	0	0	0	0	10	0	1.495	
62.5	5	4	4	4.33	10	43.3	1.796	
125	7	8	8	7.66	10	76.6	2.097	112.36
250	9	10	9	9.33	10	93.33	2.398	
500	10	10	10	10	10	100	2.699	
